# Single-cell molecular signature of pathogenic T helper subsets in type 2–associated disorders in humans

**DOI:** 10.1172/jci.insight.177720

**Published:** 2024-04-08

**Authors:** Pedro H. Gazzinelli-Guimaraes, Brittany Dulek, Phillip Swanson, Justin Lack, Mario Roederer, Thomas B. Nutman

**Affiliations:** 1 Laboratory of Parasitic Diseases,; 2 Integrated Data Science Services, and; 3 Vaccine Research Center, National Institute of Allergy and Infectious Diseases, NIH, Bethesda, Maryland, USA.

**Keywords:** Immunology, Infectious disease, Adaptive immunity, Allergy, Th2 response

## Abstract

To unravel the heterogeneity and molecular signature of effector memory Th2 cells (Tem2), we analyzed 23 individuals’ PBMCs of filaria-infected (Filaria^+^) and 24 healthy volunteers (Filaria^–^), with or without coincident house dust mite (HDM) allergic sensitization. Flow cytometry revealed 3 CD4^+^ Tem subsets — CCR4^+^CCR6^+^CRTH2^–^ Tem17, CCR4^+^CCR6^-^CRTH2^+^ Tem2, and CCR6^+^CCR4^+^CRTH2^+^ Tem17.2 — markedly enriched in Filaria^+^ individuals. These subsets were sorted and analyzed by multiomic single-cell RNA immunoprofiling. SingleR-annotated Th2 cells from Tem2 and Tem17.2 cell subsets had features of pathogenic Th2 effector cells based on their transcriptional signatures, with downregulated *CD27* and elevated expression levels of *ITGA4*, *IL17RB*, *HPGDS*, *KLRB1*, *PTGDR2*, *IL9R*, *IL4*, *IL5*, and *IL13* genes. When the Filaria^+^ individuals were subdivided based on their allergic status, Tem2 cells in HDM^+^Filaria^+^ individuals showed an overall reduction in TCR diversity, suggesting the occurrence of antigen-driven clonal expansion. Moreover, HDM^+^Filaria^+^ individuals showed not only an expansion in the frequency of both Tem2 and Tem17.2 cell subsets, but also a change in their molecular program by overexpressing *GATA3*, *IL17RB*, *CLRF2*, and *KLRB1*, as well as increased antigen-induced IL-4, IL-5, and IL-13 production, suggesting that aeroallergens reshape the transcriptional and functional programming of Th2 cell subsets in human filarial infection toward a pathogenic immunophenotype.

## Abstract 2

Environmental allergens reshape the transcriptional and functional programming of Th2 cell subsets towards a pathogenic immunophenotype in human filarial infection.

## Introduction

Strong type 2 immune responses are associated with helminth infections, but also drive allergic disorders (1, 2). A variety of studies have suggested that helminth infection can both positively and negatively affect allergic diseases (3). Epidemiological evidence indicates that chronic exposure to helminth parasites diminishes the development of allergic diseases, which is the basis of the hygiene hypothesis (4–7). However, it has also been shown that certain parasitic nematodes (e.g., *Ascaris* spp. or *Anasakis simplex*) can drive atopic disorders (8) and can aggravate the clinical symptoms of asthma (9, 10).

Not only are allergic diseases increasing dramatically worldwide, but, because of marked population movements that have come from globalization, helminth infection and allergic diseases have intersected with much greater frequency than heretofore noted, with nearly half of the world’s population either harboring helminth parasites or suffering from allergic diseases (11, 12). Thus, it is important to understand the immunological interplay between these 2 major public health problems.

Memory CD4^+^ T cells, including Th2 effector cells, are key components in the pathogenesis of allergic diseases and helminth infections (13–16). Th2 cells and their signature cytokines (especially IL-4) are crucial for human B cell maturation and class-switching to the IgE and IgG4 isotypes (17–19), as well for the recruitment eosinophils to the tissues (IL-5 and IL-13) (16) and activation of mast cells and basophils (7). Over the last decade, several investigators have explored the heterogeneity and functionality of distinct memory Th2 cell subsets, including their capacity to produce large amounts of cytokines and to differentiate into a vast repertoire of identities (14, 15, 20–23). Recently, Mitson-Salazar et al. (23), studying patients with eosinophilic gastrointestinal disease, characterized a novel Th2 effector cell subset, coexpressing CRTH2, hPGDS, and CD161 with innate-like responsiveness and enhanced effector function that was highly associated with allergic eosinophilic inflammatory disease. Later, Wambre and collaborators (24) demonstrated that this new subpopulation of pathogenic Th2 effector cells comprise the majority of the allergen-specific T cells in individuals with allergies to food, pollen, pet dander, mold, or house dust mite (HDM). This pathogenic subset of memory Th2 cells (termed Th2A or Tpath2 cells) characterized by their concomitant expression of CRTH2, CD161, CD49d, ST2, IL-17RB, and a lack of CD27 expression is phenotypically, transcriptionally, and functionally distinct from conventional Th2 cells, and reflect their capacity to produce large amounts of IL-5 and IL-9. Indeed, IL-5–producing pathogenic Th2 cells have been implicated as triggers for eosinophilic inflammation in various Th2-associated disorders such as asthma (25) and atopic dermatitis (26). Therefore, it has been postulated that the heterogeneity within Th2-associated immune responses may determine different pathologic outcomes.

We have previously demonstrated that allergic sensitization coincident with the filarial infection *Loa loa* in humans induces an augmented parasite-specific type 2–dominated immune response (27). Moreover, we showed that an increase in polyfunctional Th2 cells was correlated with elevated IgE levels and high numbers of circulating activated eosinophils, suggesting that this antigen-specific T cell hyperresponsiveness drives Th2-associated inflammatory processes (27). To further elucidate the nature of these cells, we performed a comprehensive phenotypic and transcriptional analysis at single-cell resolution to interrogate the CD4^+^ T cell diversity and their contribution to immune regulation in helminth infection and/or allergic diseases. In the present study, we have shown that filarial infection drives expansion of 3 distinct circulating memory CD4^+^ T cell subsets (CCR4^+^CCR6^+^CRTH2^–^, CCR4^+^CCR6^–^CRTH2^+^, and CCR4^+^CCR6^+^CRTH2^+^). Single-cell transcriptomic analysis of these 3 subsets revealed that the latter 2 are pathogenic Th2 effector cells and are further expanded by prior allergic sensitization. T cell receptor (TCR) analysis of these 2 subsets provides evidence of clonal expansion driven by parasite and/or HDM antigens.

## Results

### Filarial infection expands the diversity of circulating effector memory CD4^+^ Th subsets.

**Figure 1:**
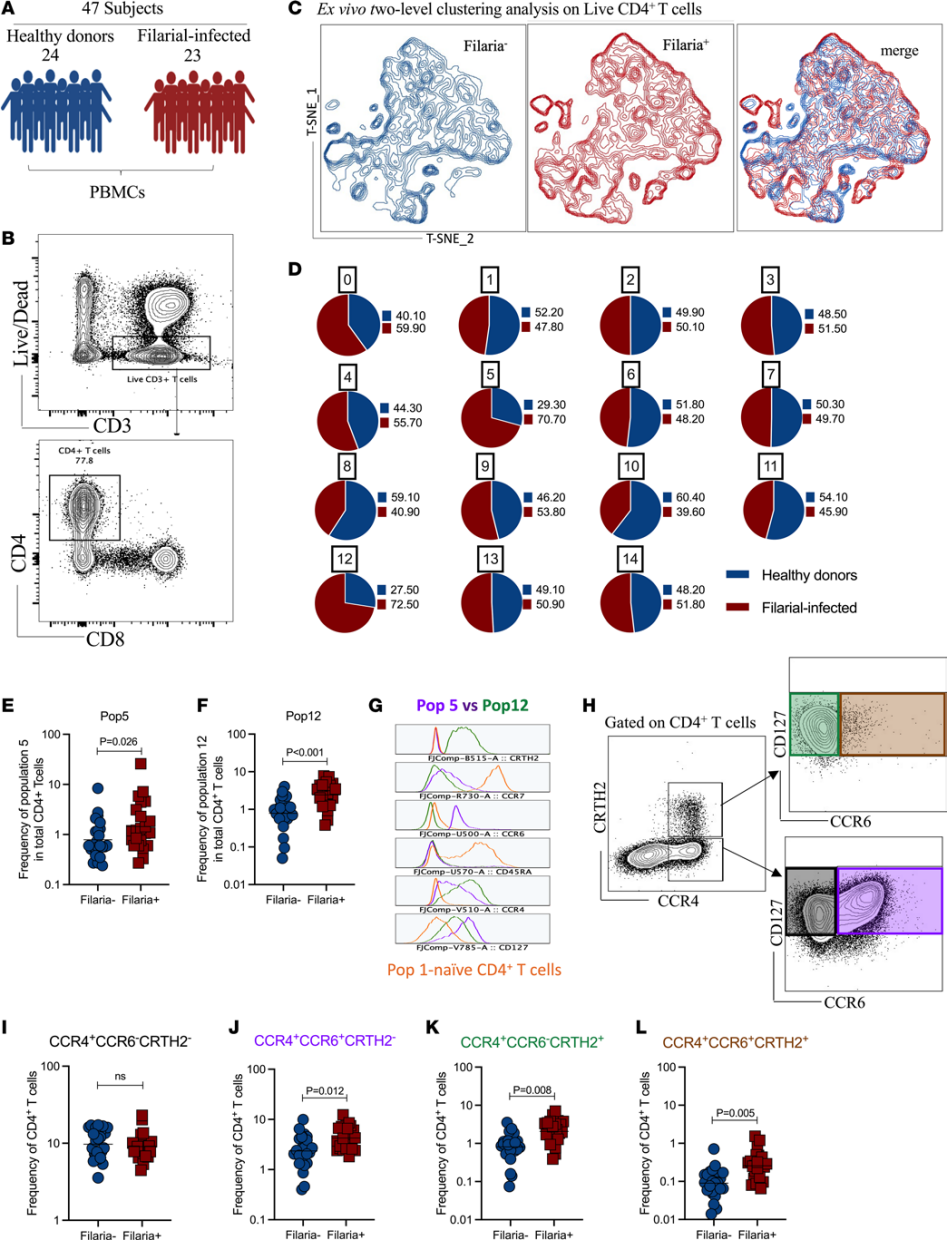
Filarial infection selectively expands subsets of circulating effector memory CD4^+^ T cells. (**A**) Schematic representation of the study summarizing the participant groups (23 filaria-infected individuals and 24 healthy donor volunteers), and gating strategies for targeting CD4^+^ T helper cells (**B**). (**C**) t-SNE plots generated by a FlowSOM analysis using a 27-color flow cytometry panel showing the high-dimensional diversity of CD4^+^ T cells among the Filaria^+^ (red) and Filaria^–^ (blue) individuals. (**D**) Proportion of filaria-infected patients’ cells and healthy donors’ cells within each cluster; note the filaria-enriched clusters, 5 and 12. Scatter plot shows the frequency of cluster 5 (**E**) and cluster 12 (**F**) deconvoluted at the individual level by groups. (**G**) Histogram plot highlighting the expression level of the markers CRTH2, CCR7, CCR6, CD45RA, CCR4, and CD127 in clusters 5 (purple) and 12 (green), in comparison with naive CD4^+^ T cells — cluster 1 (orange). (**H**) Gating strategy for the conventional flow analysis for phenotypic profiling of CD4^+^ T cells expressing CCR4^+^CCR6^–^CRTH2^–^(black), CCR4^+^CCR6^+^CRTH2^–^ (purple), CCR4^+^CCR6^–^CRTH2^+^ (green), and CCR4^+^CCR6^+^CRTH2^+^ (brown) cells. (**I**–**L**) Scatter plots show the frequency of the 4 populations described above at the individual level in their respective groups. Each dot represents a single individual, and the horizontal bars are the geometric means (GMs). Differences between the groups were considered statistically significant at *P* < 0.05 by unpaired Mann-Whitney *U* test. *P* values are indicated on each graph.

To investigate the composition and heterogeneity of CD4^+^ T cell subsets induced by filarial infection in humans, we performed immunophenotypic and functional profiling of PBMCs from 47 North American individuals: non-endemic expatriates diagnosed with filarial infection (*n* = 23) and healthy donor volunteers (*n* = 24) (Figure 1, A and B). Two-level clustering analysis of 20,000 CD4^+^ T cells from each participant revealed that filarial infection elicited changes in Th cell diversity signatures when compared with healthy controls (Figure 1C). Using a self-organizing map tool (FlowSOM), we identified 15 clusters of CD4^+^ T cells from the PBMCs of all individuals based on the expression level of 27 biomarkers used in the multiparameter flow cytometry panel (Supplemental Figure 1A; supplemental material available online with this article; https://doi.org/10.1172/jci.insight.177720DS1) and (Supplemental Table 1). When the configuration of the 15 clusters was deconvoluted by filarial infection status, 2 subpopulations of memory CD4^+^ T cell subsets, population 5 (CD45RA^–^CCR7^lo^CD127^+^CXCR3^–^CCR4^+^CCR6^+^CRTH2^–^) and population 12 (CD45RA^–^CCR7^–^CD127^+^CXCR3^–^CCR4^+^CCR6^–^CRTH2^+^) were markedly increased among the filaria-infected individuals when compared with healthy donors, based on the clustering analysis (70.70% vs. 29.30% and 72.50% vs. 27.50%, respectively; Figure 1D), as well as at the individual level (Figure 1, E and F). These 2 filaria-driven clusters represented 0.39% and 1.91% of the total numbers of CD4^+^ T cells, respectively (Supplemental Figure 1B), and were characterized based on expression level of chemokine receptors, CCR4 and CCR6, along with the expression of the prostaglandin D2 (PGD2) receptor CRTH2, and CD127 (Figure 1G). More conventional flow analysis using a combination of all these 4 markers together (Figure 1, H–L) led us to identify 2 additional other subpopulations of memory Th cells, CCR4^+^CCR6^–^CRTH2^–^ (Figure 1I) and CCR4^+^CCR6^+^CRTH2^+^ (Figure 1L), the latter being significantly increased in those with filarial infection (geometric mean [GM]: 0.25% vs. 0.08%, *P* = 0.005; Figure 1L).

### Functional heterogeneity of CD4^+^ T cell subsets promotes type 2 cytokine signatures in filarial infection.

**Figure 2:**
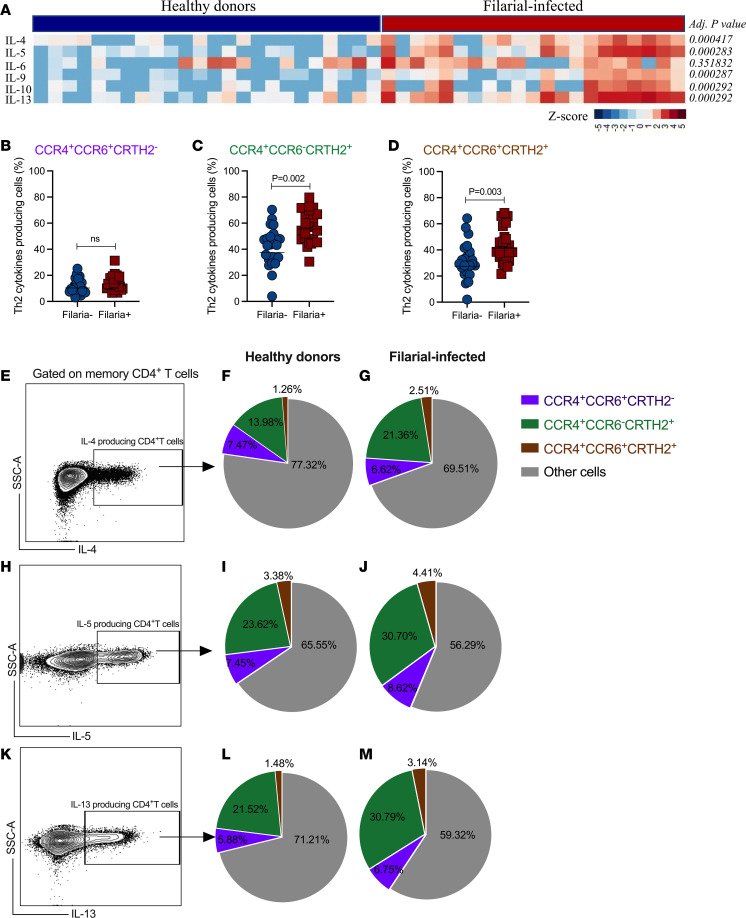
Functional diversity of CD4^+^ T cell subsets demonstrate type 2 cytokine signatures in filarial infection. (**A**) Heatmap showing the net production of IL-4, IL-5, IL-6, IL-9, IL-10, and IL-13 levels (pg/mL transformed in *z* score) in the culture supernatant after in vitro stimulation with BMA (10 μg/mL). Each column represents a single individual. The scatter plot shows the frequency of CCR4^+^CCR6^+^CRTH2^–^ (**B**), CCR4^+^CCR6^–^CRTH2^+^ (**C**), and CCR4^+^CCR6^+^CRTH2^+^ (**D**) CD4^+^ Tem cells from PBMCs of filaria-uninfected and filaria-infected patients producing IL-4, IL-5, or IL-13. Each dot represents a single individual, and the horizontal bars are the GMs. Differences between the groups were considered statistically significant at *P* < 0.05 by the Mann-Whitney *U* test. *P* values are indicated on each graph. The frequency of IL-4– (**E**), IL-5– (**H**), and IL-13–producing (**K**) CD4^+^ Tem cells by intracellular staining following 12-hour stimulation with PMA/ionomycin and brefeldin A. The proportions of each Th cell subset, CCR4^+^CCR6^+^CRTH2^–^ (purple), CCR4^+^CCR6^–^CRTH2^+^ (green), and CCR4^+^CCR6^+^CRTH2^+^ (brown), and other cells (gray) from healthy donors (**F**, **I**, and **L**) and filaria-infected patients (**G**, **J**, and **M**).

Next, we interrogated whether these expanded CD4^+^ T cell subsets associated with filarial infection had functional consequences by assessing in vitro cytokine/chemokine production following stimulation with filarial parasite *Brugia malayi* antigen (BMA). Responses measured in the culture supernatant revealed a marked type 2 cytokine signature in the filaria-infected patients, with antigen-driven levels of IL-4 (*P* < 0.0004), IL-5 (*P* < 0.0002), IL-9 (*P* < 0.0002), IL-10 (*P* < 0.0002), and IL-13 (*P* < 0.0002) when compared with supernatants of antigen-stimulated cells from healthy donors (Figure 2A). We next assessed the ability of the 3 CD4^+^ T cell subsets significantly enriched in filaria-infected patients to produce the type 2–associated cytokine IL-4, IL-5, or IL-13. The results indicated that although enriched in filarial infection, the frequencies of CCR4^+^CCR6^+^CRTH2^–^ CD4^+^ T cells from filaria-infected patients able to produce IL-4, IL-5, or IL-13 were not different when compared to the same subset from healthy controls (13.42% vs. 10.43%, *P* < 0.05; Figure 2B). In contrast, the frequencies IL-4–, IL-5–, and IL-13–producing CCR4^+^CCR6^–^CRTH2^+^ (55.5% vs. 36.6%, *P* = 0.002; Figure 2C) and CCR4^+^CCR6^+^CRTH2^+^ (41.2% vs. 27.6%, *P* = 0.022; Figure 2D) CD4^+^ T cells from filaria-infected individuals were markedly higher than in those cells from filaria-uninfected individuals.

Finally, we examined the relative contribution of these Th2 cytokine–producing CD4^+^ T cell subsets to the overall T cell–induced type 2 signature observed in the filaria-infected patients. Our analysis indicated that the overall increase in Th2 cytokine–producing cells in the filaria-infected patients relies primarily on the marked increase in IL-4– (Figure 2, E–G), IL-5– (Figure 2, H–J), and IL-13–producing (Figure 2, K–M) CCR4^+^CCR6^–^CRTH2^+^ CD4^+^ T cell subsets.

### Preexisting HDM allergic sensitization enhances both Th2 cell subsets and dynamize their function in filaria-infected patients.

**Figure 3:**
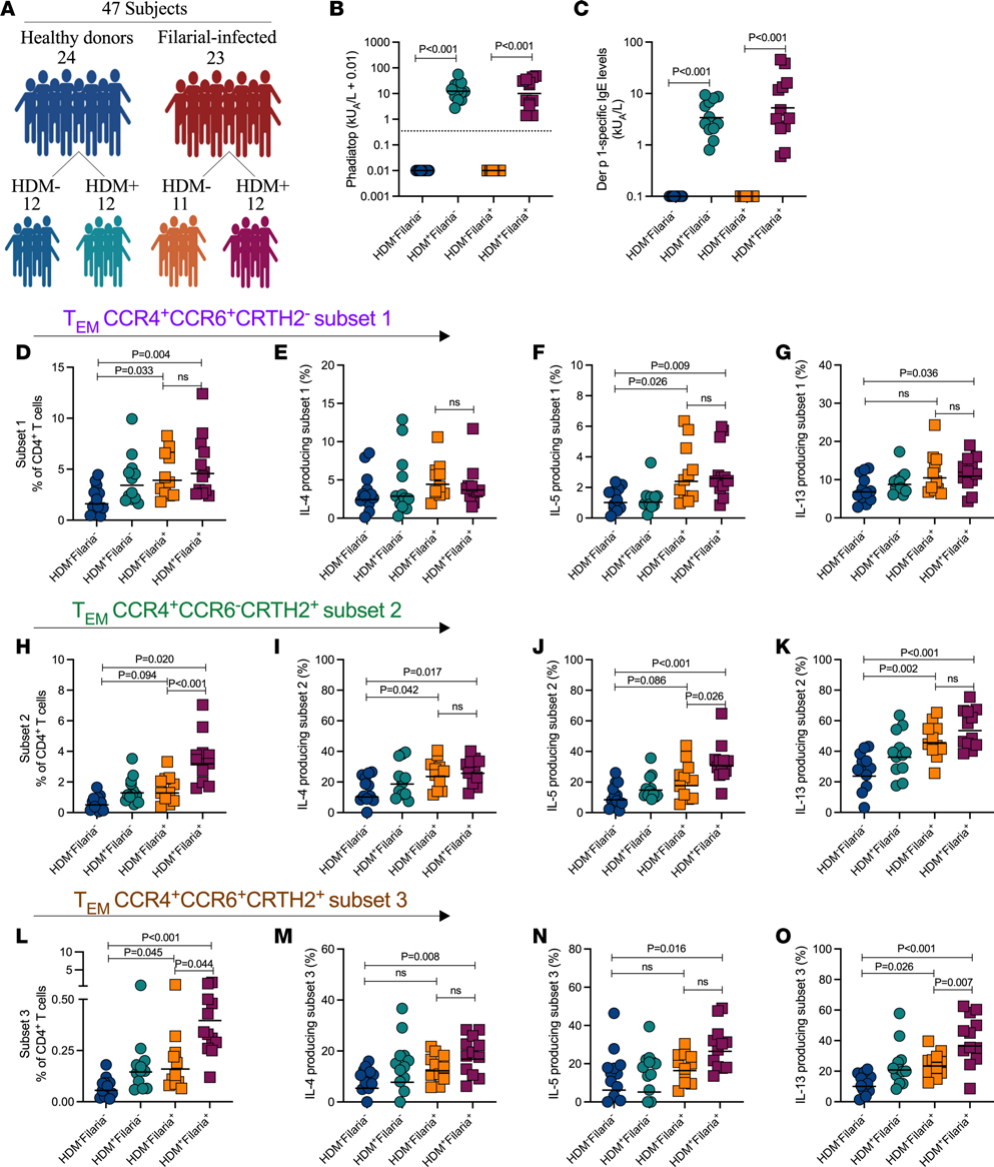
HDM allergic sensitization concomitant with filarial infection is associated with a marked increase in the frequency of both Tem2 and Tem17.2 cell subsets. (**A**) Schematic representation of the study, summarizing the participant groups according to their infection and allergic status. (**B** and **C**) ImmunoCAP Phadiatop analysis showing the allergen-specific IgE levels in each of the 4 groups. Allergen-specific IgE levels greater than 0.35 kUA/L (dotted line) were considered allergic/atopic. (**D**, **H**, and **L**) Scatter plots from ex vivo flow cytometry analysis of all 3 CD4^+^ Tem subsets at the individual level. The frequencies of IL-4– (**E**, **I**, and **M**), IL-5– (**F**, **J**, and **N**), and IL-13–producing (**G**, **K**, and **O**) CD4^+^ Tem cell subsets were also assessed for all the patients following stimulation with PMA/ionomycin in vitro. Each dot represents a single individual, and the horizontal bars are the GMs. Differences between the groups were considered statistically significant at *P* < 0.05 by Kruskal-Wallis test followed by Dunn’s multiple-comparison test. *P* values are indicated on each graph.

We next investigated whether concomitant HDM allergic sensitization changed either the frequency or function of the filaria-enriched CD4^+^ T cell subsets (Figure 3A). Individuals with allergen-specific IgE levels greater than 0.35 kilounits of antibody per liter (kUA/L) and with high levels of Der p 1–specific IgE were considered to be HDM sensitized (Figure 3, B and C).

Interestingly, the HDM allergic sensitization status played no role in changing the frequency of the CCR4^+^CCR6^+^CRTH2^–^ cells (Figure 3D) nor did it change their spontaneous production of IL-4, IL-5, and IL-13 among the filaria-infected patients (Figure 3, E–G). However, concomitant HDM allergic sensitization in concert with filarial infection was associated with a distinct increase in the frequencies of both CCR4^+^CCR6^–^CRTH2^+^ (3.19% vs. 1.28%, *P* < 0.001; Figure 3H) and CCR4^+^CCR6^+^CRTH2^+^ (0.39% vs. 0.15%, *P* = 0.044; Figure 3L) when compared with non-atopic filaria-infected patients (HDM^–^Filaria^+^). In addition, HDM sensitization induced an increase in the frequency of IL-5–producing CCR4^+^CCR6^–^CRTH2^+^ CD4^+^ T cells (30.7% vs. 17.6%, *P* = 0.026; Figure 3J), as well as in the frequency of IL-13–producing CCR4^+^CCR6^+^CRTH2^+^ CD4^+^ T cells (33.5% vs. 23.3%, *P* = 0.007; Figure 3O) in the HDM^+^Filaria^+^ group when compared with the HDM^–^Filaria^+^ group. No differences were found for IL-4– and IL-13–producing CCR4^+^CCR6^–^CRTH2^+^ CD4^+^ T cells (Figure 3, I and K) or for IL-4– and IL-5–producing CCR4^+^CCR6^+^CRTH2^+^ CD4^+^ T cells (Figure 3, M and N) when comparing the HDM^+^Filaria^+^ group with the HDM^–^Filaria^+^ group.

**Figure 4:**
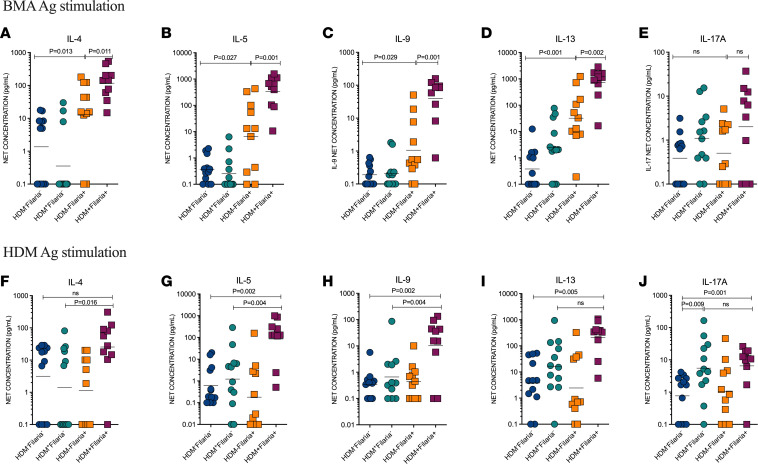
HDM allergic sensitization underlies a hyperreactive antigen-specific Th2 cytokine production in coincident filarial infection. PBMCs (2 × 10^5^ each) from participants in all 4 groups (HDM^–^Filaria^–^, *n* = 12; HDM^+^Filaria^–^, *n* = 12, HDM^–^Filaria^+^, *n* = 11, and HDM^+^Filaria^+^, *n* = 10) were stimulated in vitro for 48 hours in the absence or presence of different antigens (Ag). After incubation, net production of IL-4 (**A** and **F**), IL-5 (**B** and **G**), IL-9 (**C**–**H**), IL-13 (**D** and **I**), and IL-17A (**E** and **J**) following stimulation with BMA (10 μg/mL) (**A**–**E**) or HDM (10 μg/mL) (**F**–**J**) were calculated. Each dot represents a single individual, and the horizontal bars are the GMs. Differences between the groups were considered statistically significant at *P* < 0.05 by Kruskal-Wallis test followed by Dunn’s multiple-comparison test. *P* values are indicated on each graph.

Multiplex cytokine screening (Supplemental Figure 3) in the culture supernatant of PBMCs from all 4 groups, stimulated in the absence (media) or in the presence of parasite antigen (BMA) or HDM extract, confirmed that HDM sensitization concomitant with filarial infection drives a remarkable type 2–associated hyperreactivity, leading to a marked increase in the levels of filarial antigen–specific IL-4 (117.3 ± 3.0 pg/mL vs. 13.2 ± 13.4 pg/mL, *P* = 0.011; Figure 4A), IL-5 (336.8 ± 4.7 pg/mL vs. 6.6 ± 22.8 pg/mL, *P* = 0.001; Figure 4B), IL-9 (39.75 ± 5.4 pg/mL vs. 1.06 ± 7.95 pg/mL, *P* < 0.001; Figure 4C), and IL-13 (720.4 ± 4.3 pg/mL vs. 32.3 ± 11.6 pg/mL, *P* = 0.002; Figure 4D), but not IL-17A (Figure 4E) when compared with HDM^–^Filaria^+^ patients. Strikingly, the hyperreactivity caused by coincident allergic sensitization and filarial interaction was also evident for HDM allergen–driven responses, including higher levels of IL-4 (25.5 ± 9.1 pg/mL vs. 1.3 ± 16.7 pg/mL, *P* = 0.016; Figure 4F), IL-5 (85.4 ± 11.52 pg/mL vs. 1.2 ± 23.1 pg/mL, *P* = 0.004; Figure 4G), and IL-9 (10.2 ± 13.5 pg/mL vs. 0.6 ± 7.5 pg/mL, *P* = 0.004; Figure 4H), but not IL-13 and IL-17A (Figure 4, I and J) induced by HDM extract in the HDM^+^Filaria^+^ group when compared with HDM^+^Filaria^–^. Notably, we also observed a significant increase in the baseline levels (media-stimulated cells) of IL-5, IL-13, and IL-17A in the HDM^+^Filaria^+^ individuals when compared with both HDM^–^Filaria^+^ and HDM^+^Filaria^–^ individuals (Supplemental Figure 4, B, D, and E). While TNF-α baseline levels were increased in the HDM^–^Filaria^+^ group when compared with the HDM^–^Filaria^–^ group (Supplemental Figure 4F), no inducible TNF-α levels were observed after antigenic stimulation with either BMA or HDM in the groups (Supplemental Figure 4, H and J). Finally, although no differences in IFN-γ were found at baseline levels in the culture supernatant of all groups, HDM^+^Filaria^+^ individuals’ cells stimulated with both BMA and HDM antigens showed an increased net production when compared with the HDM^–^Filaria^–^ group (Supplemental Figure 4, I and K).

### Single-cell transcriptomic analysis identifies molecular signature of pathogenic Th2 cells among filaria-associated CD4^+^ T cell subsets.

**Figure 5:**
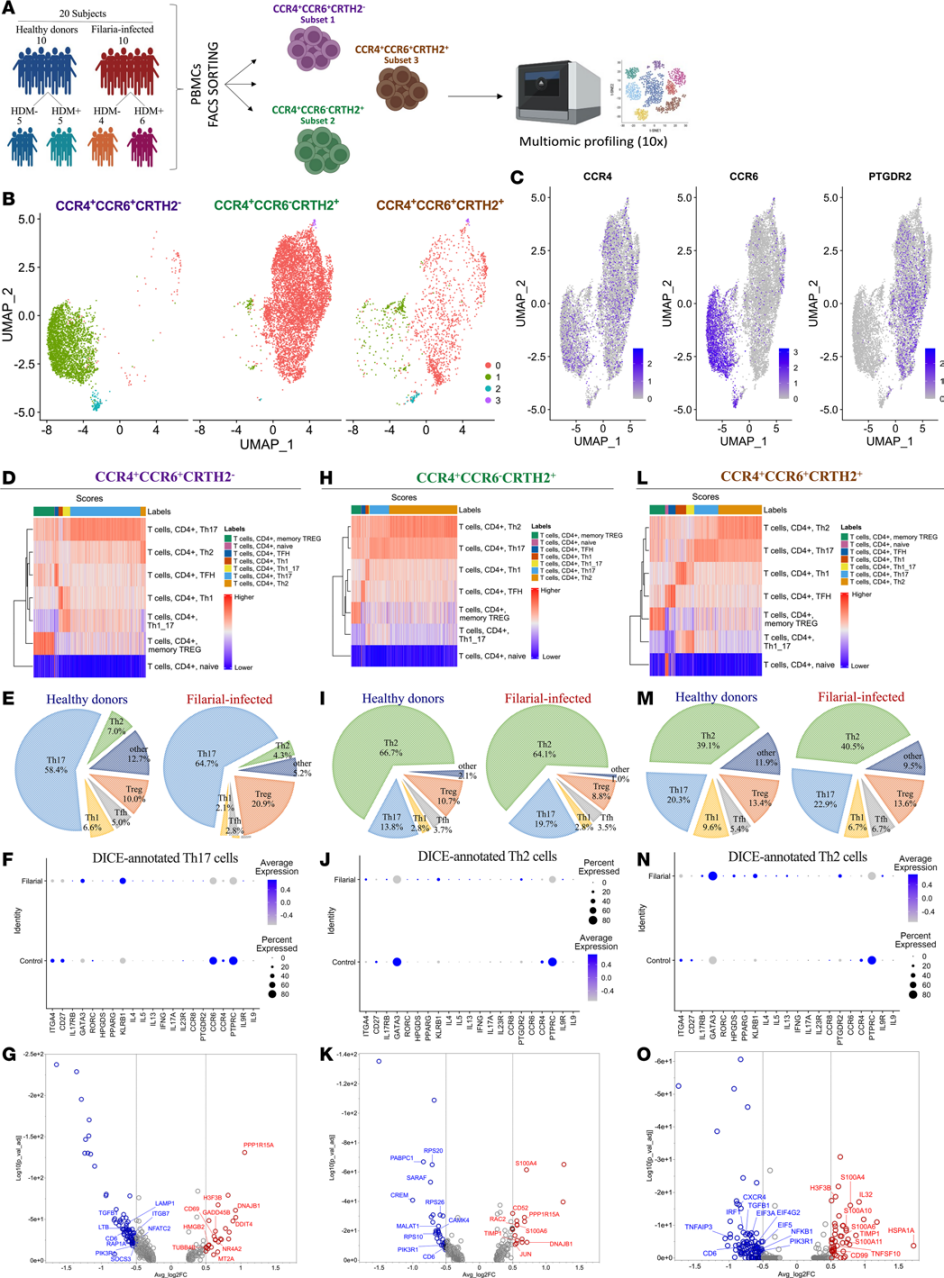
Single-cell molecular program of filaria-enriched CD4^+^ Tem cell subsets reveals a heterogeneous pathogenic repertoire. (**A**) Schematic representation of the number of individuals of each group that had live CD3^+^CD4^+^CCR7^–^CD45RO^+^ cells expressing CCR4^+^CCR6^+^CRTH2^–^ (subset 1), CCR4^+^CCR6^–^CRTH2^+^ (subset 2), or CCR4^+^CCR6^+^CRTH2^+^ (subset 3) sorted and sequenced for a single-cell multiomic immunoprofiling. (**B**) UMAP plot showing the clustering analysis of the integrated data set of 3 subsets from the 4 groups, highlighting 2 major common clusters (0 in red) and (1 in beige). (**C**) Feature plot showing the gene expression level of *CCR4*, *CCR6*, and *PTGDR2* among the major clusters of each CD4^+^ Tem subset. (**D**, **H**, and **L**) Heatmaps showing the cell type annotation within each CD4^+^ Tem subset using Database of Immune Cell Expression (DICE) based on the cell’s molecular signature. (**E**, **I**, and **M**) Proportion of different DICE-annotated cell types within each CD4^+^ Tem subset showing the heterogeneity of these subsets between healthy donors’ and filaria-infected patients’ cells. Dot plots from DICE-annotated Th17 cells from subset 1 (**F**), DICE-annotated Th2 cells from subset 2 (**J**), and DICE-annotated Th2 cells from subset 3 (**N**), highlighting the comparison in gene expression between filaria-infected patients and healthy controls, for a group of preselected genes associated with a pathogenic molecular program. Volcano plots with the most relevant differentially expressed genes in filaria-infected patients versus the controls for DICE-annotated Th17 cells from subset 1 (**G**), DICE-annotated Th2 cells from subset 2 (**K**), and DICE-annotated Th2 cells from subset 3 (**O**).

After characterizing the immunophenotype and function of the 3 CD4^+^ T cell subsets expanded in filarial infection and their further changes driven by concomitant HDM allergen sensitization, we next used multiomic single-cell RNA profiling to understand more fully their molecular nature (and heterogeneity). We separately sorted 3 populations of memory CD4^+^ T cells into those that were CCR4^+^CCR6^+^CRTH2^–^ (subset 1), CCR4^+^CCR6^–^CRTH2^+^ (subset 2), and CCR4^+^CCR6^+^CRTH2^+^ (subset 3) from PBMCs of 10 filaria-infected patients (4 HDM^–^Filaria^+^ and 6 HDM^+^Filaria^+^) and 10 healthy donor volunteers (5 HDM^+^Filaria^–^ and 5 HDM^–^Filaria^–^) randomly selected from the original cohort of 47 individuals (Supplemental Figure 2 and Figure 5A). Single-cell mRNA data from filaria-infected patients were merged using Seurat. The analysis revealed a clear breakdown into 2 large dominant clusters, clusters 0 (red) and 1 (green), with the largest distinction between subsets 2 and 1, respectively, while subset 3 is split between the 2 main clusters (Figure 5B). To validate the sorting and purity of each subset, we analyzed the expression of *CCR4*, *CCR6*, and *PTGDR2* genes in the merged cells (Figure 5C).

Cells from subsets 1, 2, and 3 from both filaria-infected patients and healthy donor volunteers were annotated using the Database of Immune Cell Expression (DICE). Interestingly, the Spearman’s coefficient correlation–based annotation revealed a marked heterogeneity within each cluster, suggesting that cell identification by phenotypic analysis alone has important limitations in cell subset-type classification. DICE fine annotation revealed that CCR4^+^CCR6^+^CRTH2^–^ subset 1 is largely composed of Tregs and Th17 cells, with a marked predominance of Th17 cells in both Filaria^–^ (58.4%) and Filaria^+^ (64.7%) individuals (Figure 5, D and E). In contrast, the CCR4^+^CCR6^–^CRTH2^+^ subset 2 is composed primarily of Th2 cells (Filaria^–^ = 66.7% and Filaria^+^ = 64.1%), with a small frequency of Tregs and Th17 cells (Figure 5, H and I). Indeed, this is consistent with the functional analysis (cytokine production) of filaria-enriched CD4^+^ T cell subsets, which indicated that subset 2 is the major source of type 2 cytokines, including IL-4, IL-5, and IL-13 in filaria-infected patients. Lastly, CCR4^+^CCR6^+^CRTH2^+^ subset 3 represented the population with the most diversity, with a relevant mix of Tregs, Th1, Th2, and Th17 cells. There was a slight plurality in favor of the Th2 cells in both Filaria^–^ (39.1%) and Filaria^+^ (40.5%) groups (Figure 5, L and M).

Next, we examined the molecular signature of the major populations within each cluster (Th17 cells, subset 1; Th2 cells, subset 2; and Th2 cells, subset 3). The transcriptional analysis of filaria-enriched subset 1 Th17 cells was characterized by overexpression of *CCR6*, *CXCR4*, *LGALS3*, *KLRB1*, *USP10*, and *AQP3*, when compared with both Th2 cell subsets (Supplemental Figure 5, A and B). In addition, the differential gene expression driven by filarial infection in comparison with healthy donors of subset 1 demonstrated that CCR4^+^CCR6^+^CRTH2^–^ Th17 cells from healthy donors are characterized by upregulated expression of *ITAG4*, *CD27*, *RORC*, *IL23*, *CCR6*, *CCR4*, and *PTPRC* genes, as well as remarkable expression of *PCBP1*, *BTG1*, and *GNB2*, among others (Figure 5F). The molecular signature of Th17 cells from subset 1 driven by filarial infection showed a downregulation of *RORC*, *IL23*, *ITAG4* (CD49d), and *CD27* expression, but an upregulation of *GATA3* expression and very high transcription of *KLRB1* (CD161), *IL4*, *IL5*, *IL13*, *IL17*, *CCR8*, and *IL9R* (Figure 5, F and G).

Moreover, filaria-driven Th2 cells from both subset 2 and subset 3 were characterized by a common overexpression of *RPS2*, *GATA3*, *KRT1*, *CYBA*, *SELL*, *PTGDR2*, *GNB2*, and others (Supplemental Figure 5, A and B) when compared with the Th17 cells. Interestingly, the molecular differences between the 2 subsets of Th2 cells were seen by the overexpression of *PCBP1*, *KLF2*, and *FAM107B* in the CCR4^+^CCR6^–^CRTH2^+^ subset 2 population, and the upregulation of *MTRNR2L8*, *PHLDA1*, *ROS1*, and *KLRB1* in the CCR4^+^CCR6^+^CRTH2^+^ subset 3 population (Supplemental Figure 5C). The differential gene expression driven by filarial infection in comparison with healthy donors of subset 2 (CCR4^+^CCR6^–^CRTH2^+^) revealed a suppression of the transcript levels of *CD27*, *GATA3*, *CCR4*, *PTPRC*, *CREM*, *SARAF*, and *MALAT1*, but upregulation of *ITGA4*, *IL17RB* (IL-25R), *HPGDS*, *KLRB1*, *PTGDR2* (CRTH2), *IL9R*, *IL4*, *IL5*, and *IL13* genes (Figure 5, J and K). Similarly, the Th2 cells from subset 3 (CCR4^+^CCR6^–^CRTH2^+^) driven by filarial infection showed a remarkable upregulation of *IL17RB*, *GATA3*, *HPGDS*, *KLRB1*, *IL4*, *IL5*, *IL13*, and *IL9R* expression, and a downregulation of *CD27* transcripts (Figure 5, N and O). Notably, the molecular program driven by filarial infection classifies the overwhelming majority of these Th2 cells as pathogenic Th2 effector cells.

### Concomitant aeroallergenic sensitization reshapes the molecular program of filaria-driven CD4^+^ Th2 cell subsets.

After showing that concomitant HDM allergic sensitization enhances Th2 subset frequencies and cytokine production in filaria-infected patients, we next investigated the influence of the HDM sensitization on the clonality of these Th2 populations in filaria-infected individuals. We investigated the diversity and abundance of TCRs in these cells by the frequency of α and β chain unique clonotypes, as well as the homeostatic clonal distribution and clonal overlap among the integrated CCR4^+^CCR6^+^CRTH2^–^ Th17 cells, CCR4^+^CCR6^–^CRTH2^+^ Th2 cells, and CCR4^+^CCR6^+^CRTH2^+^ Th2 cells from HDM^–^Filaria^–^, HDM^+^Filaria^–^, HDM^–^Filaria^+^, and HDM^+^Filaria^+^ individuals. Supplemental Table 2 contains the unique clonotype count comparisons for both the α and β chains combined among the 4 groups for the different subsets.

**Figure 6:**
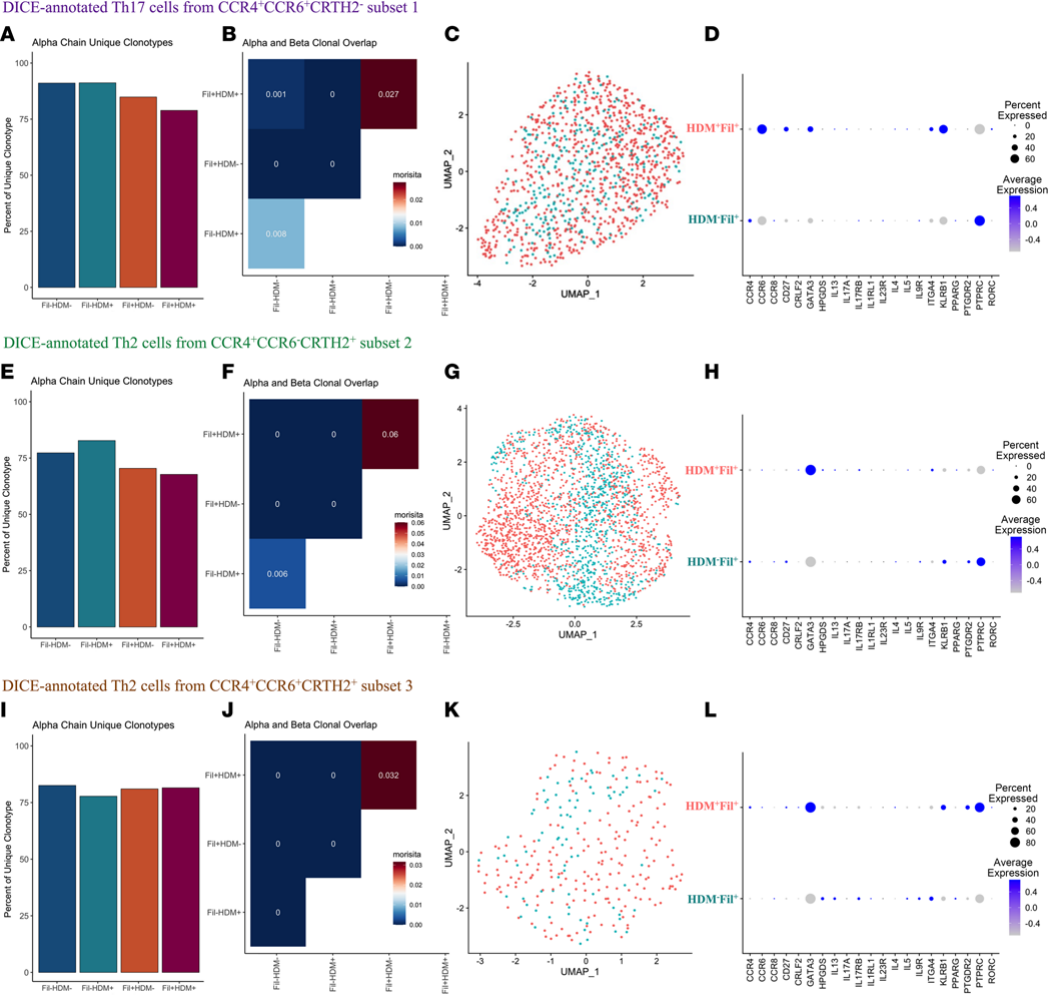
Influence of allergic sensitization in the clonotype diversity and molecular program of CD4^+^ Tem cell subsets from filaria-infected patients. VDJ genes within the DICE-annotated Th17 cells from the CCR4^+^CCR6^+^CRTH2^–^ subset, DICE-annotated Th2 cells from the CCR4^+^CCR6^–^CRTH2^+^ subset, and DICE-annotated Th2 cells from CCR4^+^CCR6^+^CRTH2^+^ subset were analyzed in each group through the assessment of the unique clonotype percentages for the α and β chains (**A**, **E**, and **I**), and the clonal overlap using the Morisita Index of dispersion (**B**, **F**, and **J**). Seurat UMAP plots highlighting the clustering of CD4^+^ Tem17 cells from subset 1 (**C**), CD4^+^ Tem2 cells from subset 2 (**G**), and CD4^+^ Tem2 cells from subset 3 (**K**) from HDM^–^Filaria^+^ (blue) and HDM^+^Filaria^+^ (pink) patients, and their differentially expressed genes for a group of selected transcripts associated with pathogenic CD4^+^ Tem cell signatures (**D**, **H**, and **L**).

Interestingly, the significant decrease in the diversity of unique α chain clonotypes among both HDM^–^Filaria^+^ and HDM^+^Filaria^+^ in comparison with HDM^–^Filaria^–^ and HDM^+^Filaria^–^ groups indicated a clonal expansion for both CCR4^+^CCR6^+^CRTH2^–^ Th17 cells (Figure 6A) and CCR4^+^CCR6^–^CRTH2^+^ Th2 cells (Figure 6E), apparently driven primarily by the filarial infections. Indeed, these data were supported by the α and β clonal overlap analysis that demonstrated that the highest similarity (Morisita Index) for the TCR sequencing was between the HDM^–^Filaria^+^ and HDM^+^Filaria^+^ groups (Figure 6, B, F, and J), suggesting overlapping clones between both filaria-infected groups, independent of their atopic status. Finally, the clonal homeostatic distribution analysis demonstrated that the reduction in the diversity of CD4^+^ T cell clones driven by filarial infection was associated with a change in the clonotype group distribution from medium (0.001 < *x* ≤ 0.01) to small (1 × 10^–4^ < *x* ≤ 0.001) abundance in subset 2 (Supplemental Figure 6), suggesting that, in the filaria-infected groups, CCR4^+^CCR6^–^CRTH2^+^ Th2 cells shifted toward a less dominant clone. Although we did not observe a remarkable change in clonal diversity for subset 3 (Figure 6I), the change in the clonal distribution from large (0.01 < *x* ≤ 0.1) to medium abundance among the filaria-infected patients may indicate that a subset of CCR4^+^CCR6^+^CRTH2^+^ Th2 cell clones could be responding to a particular antigenic stimulus, causing these clones to expand and dominate the repertoire (Figure 6J).

To determine whether the molecular program of each expanded filaria-driven CD4^+^ T subset was altered by concomitant HDM sensitization, we characterized at single-cell resolution the mRNA expression of CCR4^+^CCR6^+^CRTH2^–^ Th17 cells (Figure 6, C and D), CCR4^+^CCR6^–^CRTH2^+^ Th2 cells (Figure 6, G and H), and CCR4^+^CCR6^+^CRTH2^+^ Th2 cells (Figure 6, K and L) from HDM^+^Filaria^+^ in comparison with HDM^–^Filaria^+^ individuals. Through analysis of differential gene expression and using the transcript markers associated with hyperreactive pathogenic Th2 cells, we demonstrated that coincident HDM allergic sensitization changed the transcriptional profile of both Th2 cell subsets (subsets 2 and 3) in different ways. The influence of concomitant HDM sensitization in filarial infection reshaped the molecular program of the CCR4^+^CCR6^–^CRTH2^+^ Th2 cells by upregulating the expression level of *ITAG4*, *IL17RB*, *GATA3*, *PPARG*, *HPGDS*, *CCR6*, *IL5*, and *IL13*, but by downregulating *CD27*, *KLRB1*, *IL4*, *CCR8*, *PTGDR2*, *CCR4*, and *IL9R* transcripts. The CCR4^+^CCR6^+^CRTH2^+^ Th2 cells from HDM^+^Filaria^+^, however, showed a marked upregulation of *CD27*, *GATA3*, *KLRB1*, *IL4*, *PTGDR2*, *CCR4*, and *CCR6*, but suppression of *ITAG4*, *IL17RB*, *HPGDS*, *PPARG*, *CCR8*, *IL9R*, *IL5*, and *IL-13* genes in comparison with HDM^–^Filaria^+^ cells.

In summary, our findings indicate that concurrent allergic sensitization to HDM not only amplifies the population of filaria-enriched Th2 effector cells, but also induces significant changes in their transcriptional and functional profiles, shifting them toward a more pathogenic type 2 phenotype.

## Discussion

Pathogenic type 2 responses are intricately linked to both helminth infections and allergic disorders, each representing significant global health concerns. To unravel the mechanistic basis of type 2–mediated inflammation in humans, new studies, using clinically relevant samples, are still necessary for decoding the diversity of effector CD4^+^ T cell subsets, and to understand the role played by these cells in driving a type 2 inflammation–rich environment. By employing a combination of immunophenotypic, functional, and single-cell multiomic analyses, we aimed to elucidate the heterogeneity of circulating CD4^+^ Tem cell subsets driven by helminth parasitic infections and/or allergen sensitization, including their molecular signatures, plasticity, and functional attributes.

We have previously demonstrated that allergic sensitization coincident with helminth infection exacerbates type 2–dominated immune responses (27–29). In humans, we showed that filarial parasite antigens induce a marked increase in the frequencies of circulating polyfunctional CD4^+^IL-2^+^IL-4^+^IL-5^+^TNF-α^+^ Th2 cells in the PBMCs of the group of HDM-sensitized individuals concomitantly infected with filarial parasites when compared with non-atopic, filaria-infected patients (27). In this present study, we used a variety of approaches to unravel the diversity, heterogeneity, and abundance of these cells, and we characterized the phenotype and function of CD4^+^ T cells of individuals in the context of filaria-infected individuals with or without coincident allergic sensitization. Ex vivo analysis of multiparameter flow cytometry data by a 2-level clustering tool identified 3 subsets of CD4^+^ T cells that were markedly enriched among the filaria-infected patients in comparison with healthy controls. Moreover, we showed that allergic sensitization, particularly to HDM allergens, enhances even further the expansion and function of these subsets in filaria-infected individuals, an expansion, we believe, leading to marked increases in parasite antigen- and allergen-driven cytokine production. This finding supports the idea that interactions between helminth infections and allergic sensitization can result in complex immune responses that go beyond simple additive effects. Phenotypically, these subsets shared a common lack of expression for both CCR7 and CD45RA and were distinguished among themselves by their varied expression of CCR4, CCR6, and the prostaglandin D2 receptor, CRTH2.

Typically, based on the expression of CD45RA and CCR7, human T cells can be divided into 4 subsets: CD45RA^+^CCR7^+^ naive (Tn), CD45RA^−^CCR7^+^ central memory (Tcm), CD45RA^−^CCR7^−^ Tem, and CD45RA^+^CCR7^−^ effector memory re-expressing CD45RA T (Temra) cells (30, 31). Our data in the present study suggested that filarial infection was expanding subsets of CD4^+^ Tem cells. In addition, the expression pattern of 4 surface receptors, including chemokines and CRTH2, has been used for the identification of CD4^+^ Tem subsets, which have been defined as Tem1 (CCR6^–^CXCR3^+^CCR4^+^CRTH2^–^) (32), Tem2 (CCR6^–^CXCR3^–^CCR4^+^CRTH2^+^) (33, 34), Tem17 (CCR6^+^CXCR3^–^CCR4^+^CRTH2^–^), and a unique subset that exhibits both Th17 and Th1 features, referred to as Tem17.1 (CCR6^+^CXCR3^+^CCR4^–^CRTh2^–^) (35, 36). In addition to the expression of surface markers, transcription factors that control the differentiation and function of these CD4^+^ T cell subsets have been identified, including GATA3, which appears to be critical for Tem2 cell differentiation (37), T-bet for Tem1 (38), and RORγt for Tem17 (39).

Here, we phenotypically identified the first subset significantly enriched among filaria-infected individuals as CD4^+^CD45RA^–^CCR7^–^CD127^+^CXCR3^–^CCR4^+^CCR6^+^CRTH2^–^ Tem17 cells. Babu and collaborators (40) demonstrated that the expression of the Th17 cytokines IL-17A, IL-17F, IL-21, and IL-23 was significantly upregulated in filaria-infected patients with lymphedema, suggesting a role for Th17 cells in the pathogenesis of filaria-induced pathology. However, more comprehensive characterization of the effects of these cells on the course of helminth infections had not been previously studied carefully.

Filaria-enriched subset 2 was characterized as CD4^+^CD45RA^–^CCR7^–^CD127^+^CXCR3^–^CCR6^–^CCR4^+^CRTH2^+^ Tem2 cells. Notably, additional functional analysis demonstrated that this subset was able to produce copious amounts of IL-4, IL-5, and IL-13, perhaps arguing that these cells are one of the most important sources of type 2–associated cytokines in filaria-infected patients (as seen in Figure 2). Indeed, the identification of Th2 cell hyperresponsivenes in helminth infections in humans and animal models has been well documented (28, 29). Since the 1990s, there has been shown an increased frequency of circulating populations of antigen-specific IL-4– and IL-5–secreting Th2 cells in humans infected with helminth parasites (41–43). Moreover, it has been shown that a diminished parasite-specific CD4^+^ T response is associated with an immunologic tolerance and subclinical disease in lymphatic filariasis (44). Similarly, Yazdanbakhsh et al. (45) showed that high T cell proliferative responses associated with elevated specific IgE levels have a critical role in filaria-driven pathology. Notwithstanding, in the present study, we have shown that allergic presensitization to HDM in the face of filarial infection drives not only expansion of CCR4^+^CCR6^–^CRTH2^+^ Tem2 cells, but also these cells’ ability to produce IL-5. This suggests that there may be a role for this particular T cell subset in driving pathology in both helminth infections and allergic disease. Indeed, in several studies on allergic diseases, the influx of hyperreactive IL-5–producing Tem2 populations (termed PeTh2) was crucial in driving pathologic eosinophilic inflammation in chronic asthma (46) and in chronic skin disorders (26).

Finally, the third subset found to be expanded by the filarial infection exhibited both Th17 and Th2 features (CD4^+^CD45RA^–^CCR7^–^CD127^+^CXCR3^–^CCR4^+^CCR6^+^CRTH2^+^), suggesting a subset of Tem17.2 that are referred to in the literature as dual-positive Th2/Th17 cells. Of note, although dual-positive Th2/Th17 cells, expressing both CRTH2 and CCR6, have been found in higher frequencies in the peripheral blood and BAL from asthmatic patients and have been associated with exacerbation of chronic allergic asthma (14, 47), to our knowledge this is the first report of expansion of CCR4^+^CCR6^+^CRTH2^+^ Tem17.2 cells in the peripheral blood of helminth-infected patients. It appears that these Tem17.2 cells develop from more plastic Th2 cells, in that the Th2 cells acquire the ability to produce IL-17 (15, 22), the emergence of which is dependent on the presence of Th17-inducing cytokines, including IL-1β, IL-6, and IL-21 (14).

Remarkably, one of the key contributions of the present study was the comprehensive investigation at single-cell resolution of the molecular profiles of these 3 CD4^+^ T cell subsets. Filarial infection was associated with not only an expansion of CD4^+^ Tem subsets, but also with relevant changes in their molecular program. Moreover, the differential expression analysis demonstrated that the coincident allergic sensitization with filarial infection reshaped even further the transcriptional and functional programming of CD4^+^ T cells toward a pathogenic Th2 immunophenotype. Indeed, CCR4^+^CCR6^–^CRTH2^+^ Th2 cells in HDM allergic and filaria-infected patients were associated with a downregulation of *CD27* and upregulation of *ITAG4*, *IL17RB*, *GATA3*, *PPARG*, *HPGDS*, *CCR6*, *IL5*, and *IL13*. In humans, pathogenic Th2 cells have been characterized in PBMCs from patients with eosinophil-associated gastrointestinal disorders and atopic dermatitis as CD4^+^CRTH2^hi^CD161^hi^hPGDS^hi^ T cells (reviewed in ref. 15).

Of note, based on our results from the gene set enrichment analysis in each subset (seen in Figure 5), it became clear that a classification based on surface markers is limited and not sufficient to determine the cell types. Maintaining the same phenotype these can employ different functions, depending on the microenvironment and immunological landscape.

This study aimed to understand not only the molecular nature of these pathogenic CD4^+^ T cell subsets, but also their diversity and specificity. A decrease in the frequency of unique clonotypes among the filaria-enriched CD4^+^ T subsets could indicate a reduction in the overall diversity of Tem2 cell clones, indicating that filarial infection was associated with outgrowth of several clonotypes. The shift to a smaller homeostatic distribution might suggest that a group of Tem2 clones is responding to a particular antigenic stimulus, causing these clones to expand and dominate the repertoire. Identification of those antigens/allergens that are driving such responses will be a subsequent priority.

Taken together, these refined molecular and functional profiles of CD4^+^ Tem cell subsets from individuals concurrently infected with filarial parasitic infections and sensitized to environmental allergens provide a comprehensive immunologically focused analysis of the interplay between helminth infection, allergen sensitization, and the heterogeneity of CD4^+^ T cell responses. In so doing, our data suggest that preexisting allergic sensitization reshapes the transcriptional and functional coding of CD4^+^ T cells in human filarial infection toward a more pathogenic Th2 program. This study has shed light on the Th2 effector cell diversity and their contribution to the immunopathogenesis of type 2–associated disorders and provides insights into heterogeneity and plasticity of CD4^+^ T cell subsets.

## Methods

### Study population.

For this study, sex was not considered as a biological variable. A previously described cohort was used to characterize the nature of antibody and the T cell response induced by parasite antigens and aeroallergens in the context of filaria-infected individuals with or without coincident allergic sensitization (27, 48). Twenty-one of the 23 filaria-infected patients were temporary residents of filaria-endemic countries and were definitively diagnosed with *Loa loa*, *Wuchereria bancrofti*, or *Onchocerca volvulus* infection based on parasitological and/or molecular assays. Although the time of infection is impossible to determine, the time from the earliest possible exposure to the time of diagnosis ranged from 2 months to 3 years. Moreover, 24 healthy volunteers (NIH blood bank donors) comprised the control group and had similar age ranges and ethnic background distributions to those with filarial infection. All individuals were subdivided into 2 groups (atopic or non-atopic) based on ImmunoCAP Phadiatop assay greater than 0.35 kUA/L and Der p 1–specific IgE levels greater than 0.35 kUA/L. IgE levels specific to other aeroallergens, including Timothy grass pollen, German cockroach, cat dander, and *Alternaria alternata* were also assessed (ImmunoCAP Specific IgE, Thermo Fisher Scientific). The study population therefore was as follows: group 1, HDM^–^Filaria^–^ (*n* = 12); group 2, HDM^+^Filaria^–^ (*n* = 12); group 3, HDM^–^Filaria^+^ (*n* = 11); and group 4, HDM^+^Filaria^+^ (*n* = 12) (Supplemental Table 2).

### Multiparameter flow cytometry.

Comprehensive immunophenotypic characterization was performed on 1 × 10^6^ PBMCs of all 47 individuals at baseline (media alone), or after stimulation with PMA/ionomycin (0.5/0.05 pg/mL; Sigma-Aldrich) for 12 hours, in the presence of brefeldin A (10 μg/mL; Sigma-Aldrich), by extra- and intracellular staining using a 27-multiparameter flow cytometry panel. After stimulation, cells were stained for viability (Live/Dead fixable blue, Molecular Probes) for 10 minutes in the dark at room temperature, washed with FACS buffer, and then incubated with a fluorochrome-conjugated antibody’s master mix for surface markers (Supplemental Table 1) for 30 minutes in the dark at room temperature. The cells were next washed twice with FACS buffer, and then fixed and permeabilized using a Fix/Perm buffer kit (BioLegend) for 30 minutes in the dark at 4°C. The cells were washed twice with Perm buffer (BioLegend) and resuspended with fluorochrome-conjugated antibody’s master mix for intracellular markers (Supplemental Table 1) for 30 minutes in the dark at 4°C. Finally, the cells were washed twice with Perm buffer and then acquired using the LSRFortessa X50 flow cytometer (BD Biosciences) and FACSDiva software (BD Biosciences) for acquisition. All analyses were performed using FlowJo v.10.5.3.

### In vitro culture and cytokine production.

PBMCs (2 × 10^5^) from all individuals were cultured in 200 μL of supplemented RPMI media alone, or in addition to 10 μg/mL filarial parasite antigen BMA (crude extract from the human filariid *Brugia malayi* adult worms [which have been extensively employed to activate human PBMCs in studies focusing on filarial infections due to its substantial proteomic similarity with other filarial parasites]), or with 10 μg/mL HDM (*Dermatophagoides pteronyssinus* extract; Greer Lab), or with PMA/ionomycin (0.5/0.05 pg/mL) for 48 hours in 5% CO_2_ at 37°C. After incubation, the culture supernatant was used for cytokine quantification by a 42-plex Luminex assay (Milliplex, Millipore) in accordance with the manufacturer’s recommendations.

### Sample preparation and single-cell RNA-seq libraries for next-generation sequencing.

Multiomic single-cell immune profiling was performed in 3 subsets of memory CD4^+^ T cells enriched among filaria-infected patients. Briefly, from PBMCs of 10 filaria-infected patients (4 HDM^–^Filaria^+^ and 6 HDM^+^Filaria^+^) and 10 healthy donor volunteers (5 HDM^+^Filaria^–^ and 5 HDM^–^Filaria^–^), 3 subsets of live CCR7^–^CD45RO^+^ CD4^+^ Tem cells were sorted (see gating strategy in Supplemental Figure 2 and Supplemental Table 3), including CD4^+^ T cells expressing CCR4^+^CCR6^+^CRTH2^–^ (subset 1), CCR4^+^CCR6^–^CRTH2^+^ (subset 2), and CCR4^+^CCR6^+^CRTH2^+^ (subset 3) (Figure 5). Approximately 1 × 10^4^ sorted cells of each subset from all patients were hashtagged separately with TotalSeq-C anti-human hashtag (BioLegend). Next, each set of sorted cells from individual patient cells were pooled for a total of 10,000 cells per sample, generating a total of 6 samples. Approximately 1 × 10^4^ multiplexed cells (1000 cells per patient for each sorted subset) were then loaded into the Chromium Next GEM Chip G (10× Genomics). In total, 12 libraries were made in accordance with the Chromium Single Cell V(D)J Reagent Kits User Guide (v1.1) with Feature Barcoding technology, including 6 GEX libraries and 6 VDJ-enriched libraries.

### Single-cell RNA-seq.

The resultant 12 libraries were sequenced on a NovaSeq 6000 S2 flow cell, with 26 cycles and 91 asymmetrically run cycles. FASTQ files and count matrices were created using 10× Genomics Cell Ranger v5.0.0 default parameters with the GRCh38 transcriptome reference (GRCh38-2020-A) and V(D)J reference (vdj_GRCh38_alts_ensembl-5.0.0). Quality filtering and analysis were performed using Seurat v4.1.3 (48). Seurat filtering for the 6 samples removed cells with a gene count of less than 200 or greater than 3000 and mitochondrial gene expression greater than 15%. Demultiplexing of hashtags was performed using HTOdemux from Seurat. Healthy donor and filaria-infected samples in the absence or concomitant presence of HDM allergic sensitization for each subset were integrated and visualized using uniform manifold approximation and projection (UMAP) dimension reduction using 20 principal components (PCs) and a cluster resolution of 0.1. SingleR v2.0.0 (49) was used to annotate and filter cells using fine markers from DICE (50). Differential expression analysis of subsets was performed using model-based analysis of single-cell transcriptomics (MAST) (51). Analysis of V(D)J data for each subset was performed using scRepertoire v1.8.0 (52), where clonotype was defined as the amino acid sequence. Unique clonotype counts for the α chain were compared using Wilcoxon’s signed-rank test and the Benjamini-Hochberg method to control for the false discovery rate (Supplemental Data Sets 1–3). Single-cell RNA-seq data sets for CD4^+^ Tem cell subsets from healthy donor and filaria-infected samples are available in the NCBI Gene Expression Omnibus (GEO GSE240865).

### Statistics.

All statistical analyses were performed using Prism 9.0 for Macintosh (GraphPad Software). Unless stated otherwise, GMs and the upper 95% confidence intervals of GMs (between parentheses) were used as measures of central tendency. A nonparametric Mann-Whitney *U* test was used for the analysis comparing data from filaria-infected patients versus healthy donor volunteers. A Kruskal-Wallis 1-way ANOVA followed by Dunn’s multiple-comparison test was used to evaluate the statistical differences for the subset’s frequency, for the cytokine production by the different groups, and for the frequency of CD4^+^ T cells producing cytokines. Net production of cytokines (in pg/mL) and net frequency (percentage) of CD4^+^ T cells producing cytokines were calculated by subtracting the baseline level from the level following stimulation. The differences were considered statistically significant when the corrected *P* values were less than 0.05.

### Study approval.

All individuals (and all samples collected) were part of registered protocols approved by the Institutional Review Boards of the National Institute of Allergy and Infectious Diseases (NCT00001230 and NCT00001345) for the filaria-infected individuals, and by the Department of Transfusion Medicine, Clinical Center, NIH (IRB 99-CC-0168) for the healthy donors. Written informed consent was obtained from all participants.

### Data availability.

Values for all data points in graphs are reported in the Supporting Data Values file in the supplemental material.

## Author contributions

PHGG and TBN conceptualized the study. PHGG, BD, PS, and MR developed the methodology. PHGG, PS, BD, and JL analyzed data. PHGG and PS acquired data. PHGG, PS, BD, and JL generated figures. MR and TBN acquired funding. PHGG provided project administration. TBN supervised the study. PHGG and TBN wrote the original draft of the manuscript, which was reviewed and edited by PHGG, BD, PS, JL, MR, and TBN.

## Supplementary Material

Supplemental data

Supplemental data set 1

Supplemental data set 2

Supplemental data set 3

Supporting data values

## Version 1 04/08/2024

Electronic publication
