# Supplementary material for: Single-cell molecular signature of pathogenic T helper subsets in type 2–associated disorders in humans
Source: JCI Insight. 2024 Apr 8;9(7):e177720. doi: 10.1172/jci.insight.177720 (PMC11128205; doi:10.1172/jci.insight.177720)
Supplement: Supplemental data [file jciinsight-9-177720-s156.pdf]

Supplemental materials:

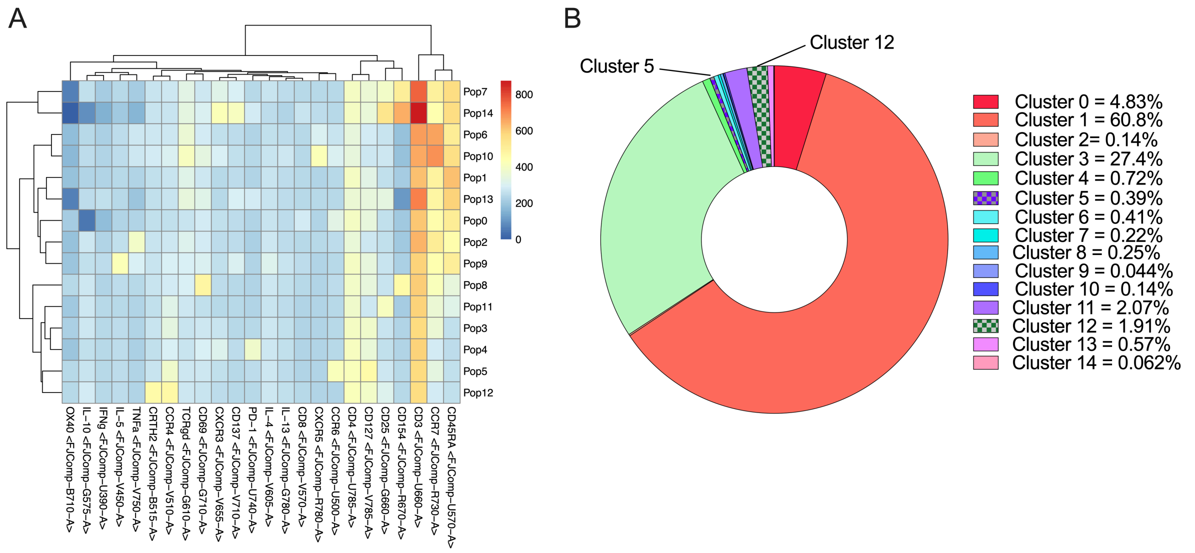

Supplemental Fig. 1. CD4$^+$ T cells subsets' relative abundance and clustering characterization. (A) Heat map showing the expression level (MFI) of 24 parameters in the 15 clusters of concatenated CD4$^+$ T cells from both filarial-infected patients and healthy donor volunteers identified by the FlowSOM analysis. (B) Proportion of each cluster from total CD4$^+$ T cells.

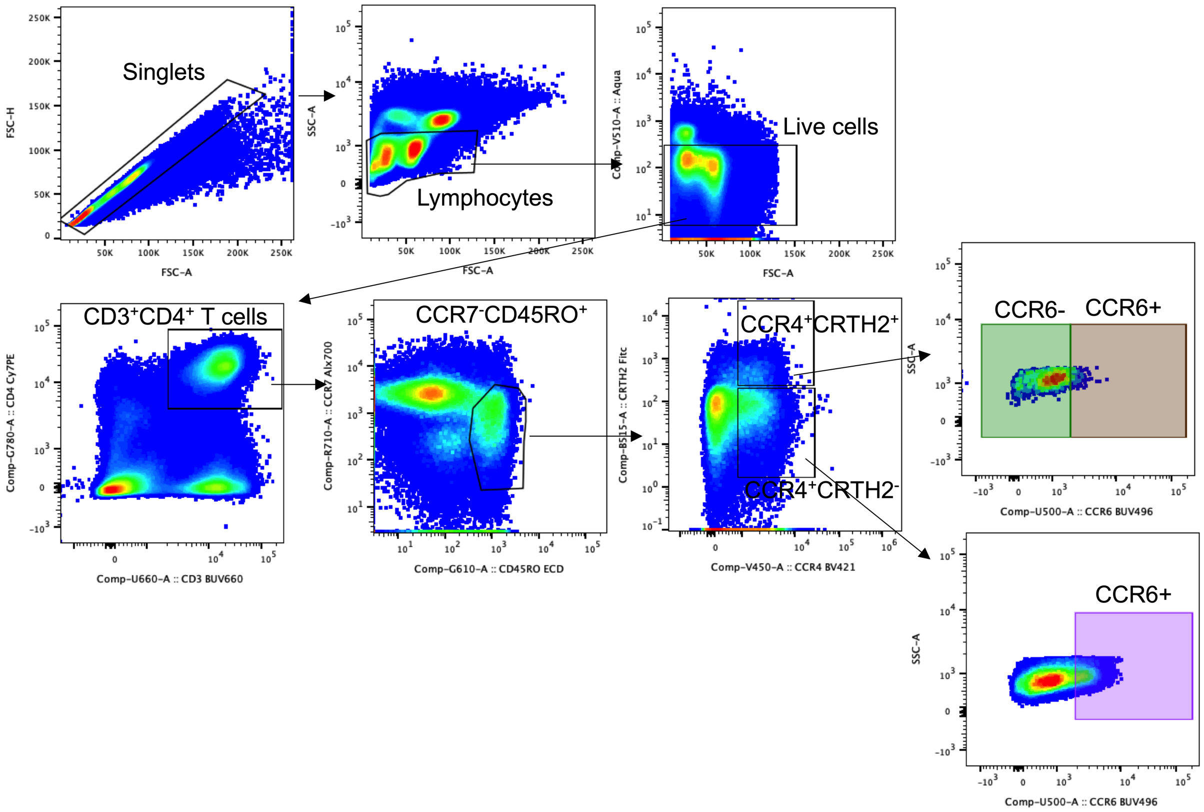

Supplemental figure 2: Phenotyping Flow Cytometry for sorting. Singlets were gated followed by subsequent lymphocytes/mononuclear cell gating by FSC-A vs SSC-A. Live cells were gated as Aqua/BV510-. Cells were then gated as CD3/BUV660+, CD4/PE-Cy7+. CD4+ T cells were further gated as CCR7/AlexaFluor700- and CD45RO/ECD+. Effector memory CD4+ T cells were further subdivided into either CCR4/BV421+CRTH2/FITC+ or CCR4/BV421+CRTH2/FITC- populations. Finally, CCR6/BUV496 expression was further gated to set the BD FACSymphony™ S6 Cell Sorter to allow for sorting the three population of interest: subset 1 (CCR4+CCR6+CRTH2-) in purple, subset 2 (CCR4+CCR6-CRTH2+) in green, and subset 3 (CCR4+CCR6+CRTH2+) in brown.

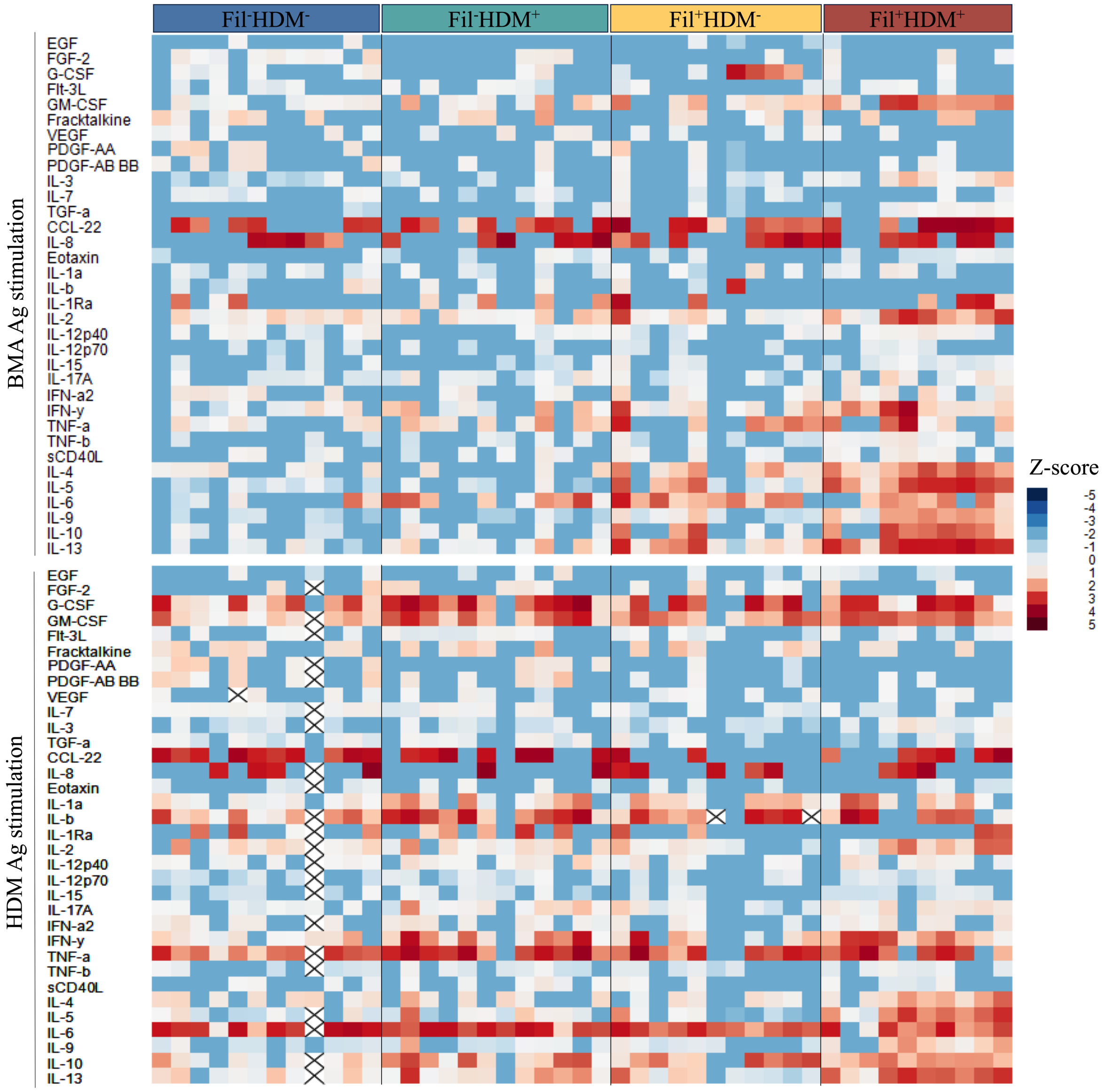

Supplemental figure 3: Overall antigen-specific cytokine, chemokine and growth factors profiling. Heat map showing the summary of all net production (transformed in z score) of cytokines, chemokines and growth factors, following stimulation with BMA (10µg/mL) (top) or HDM (bottom). Each row represents a single analyte, and each column represent a subject of their respective groups (Fil$^-$HDM$^-$ n=12; Fil$^-$HDM$^+$ n=12, Fil$^+$HDM$^-$ n=11, and Fil$^+$HDM$^+$ n=10). Undetected levels were excluded from the analysis and marked with a x.

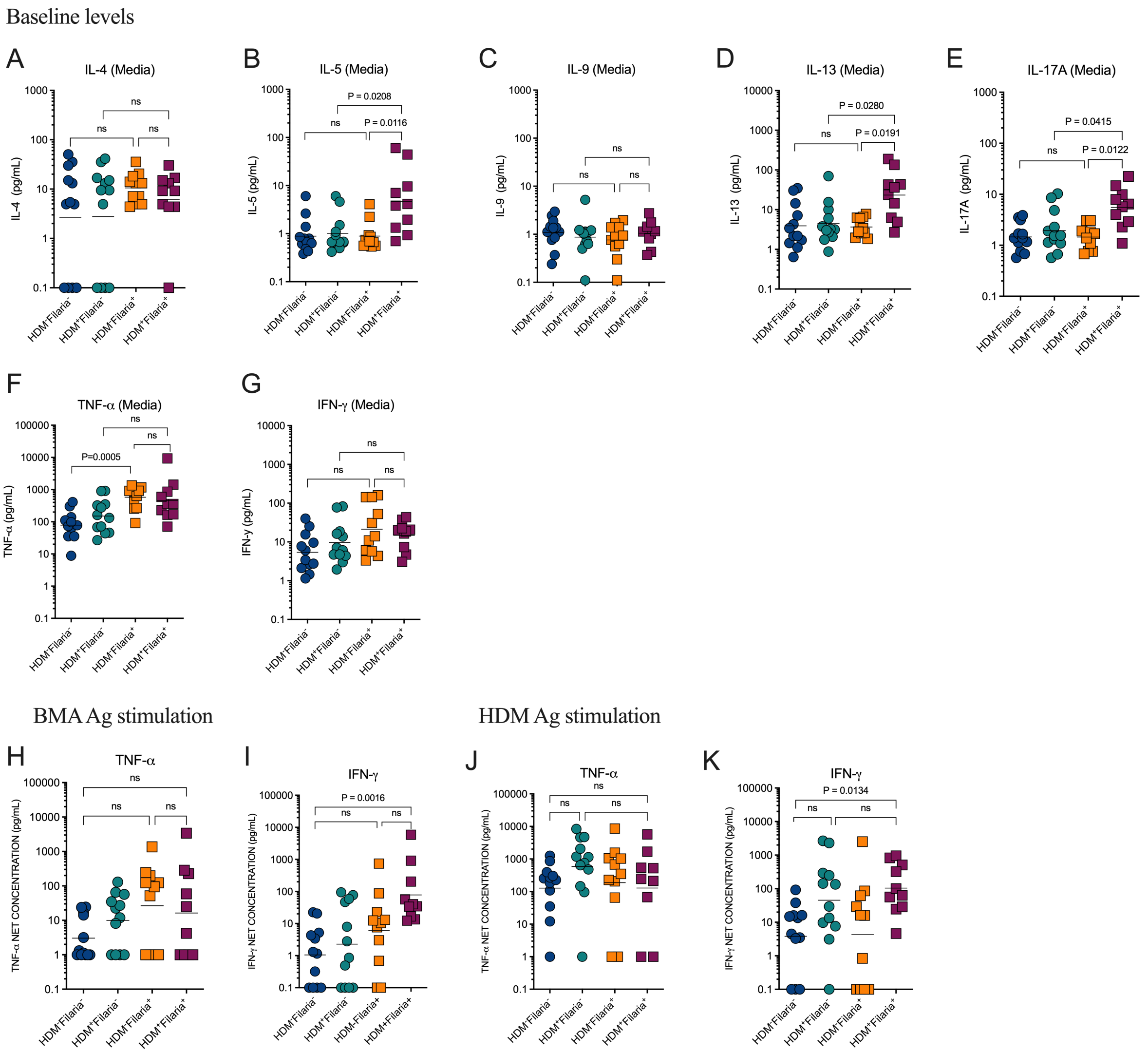

Supplemental figure 4: Baseline levels for cytokines in the culture supernatant and antigen-specific Th1-cytokine profiling. $2x10^5$ PBMCs from subjects of all four groups (Fil$^-$HDM$^-$ n=12; Fil$^-$HDM$^+$ n=12, Fil$^+$HDM$^-$ n=11, and Fil$^+$HDM$^+$ n=10) were stimulated *in vitro* for 48 hours in the absence (media-stimulated) or presence of BMA and HDM antigens. Baseline levels of IL-4, IL-5, IL-9, IL-13, IL-17A, TNF-α and IFN-γ (A-G) were quantified in the culture supernatant by a Luminex. After incubation, net production of TNF-α and IFN-γ following stimulation with BMA (10μg/mL) (H-1) or HDM (10μg/mL) (J-K) were also calculated. Each dot represents a single individual, and the horizontal bars are the GMs. Differences between the groups were considered statistically significant at $P < 0.05$ by Kruskal-Wallis test followed by Dunn's multiple-comparisons test. P values are indicated on each graph.

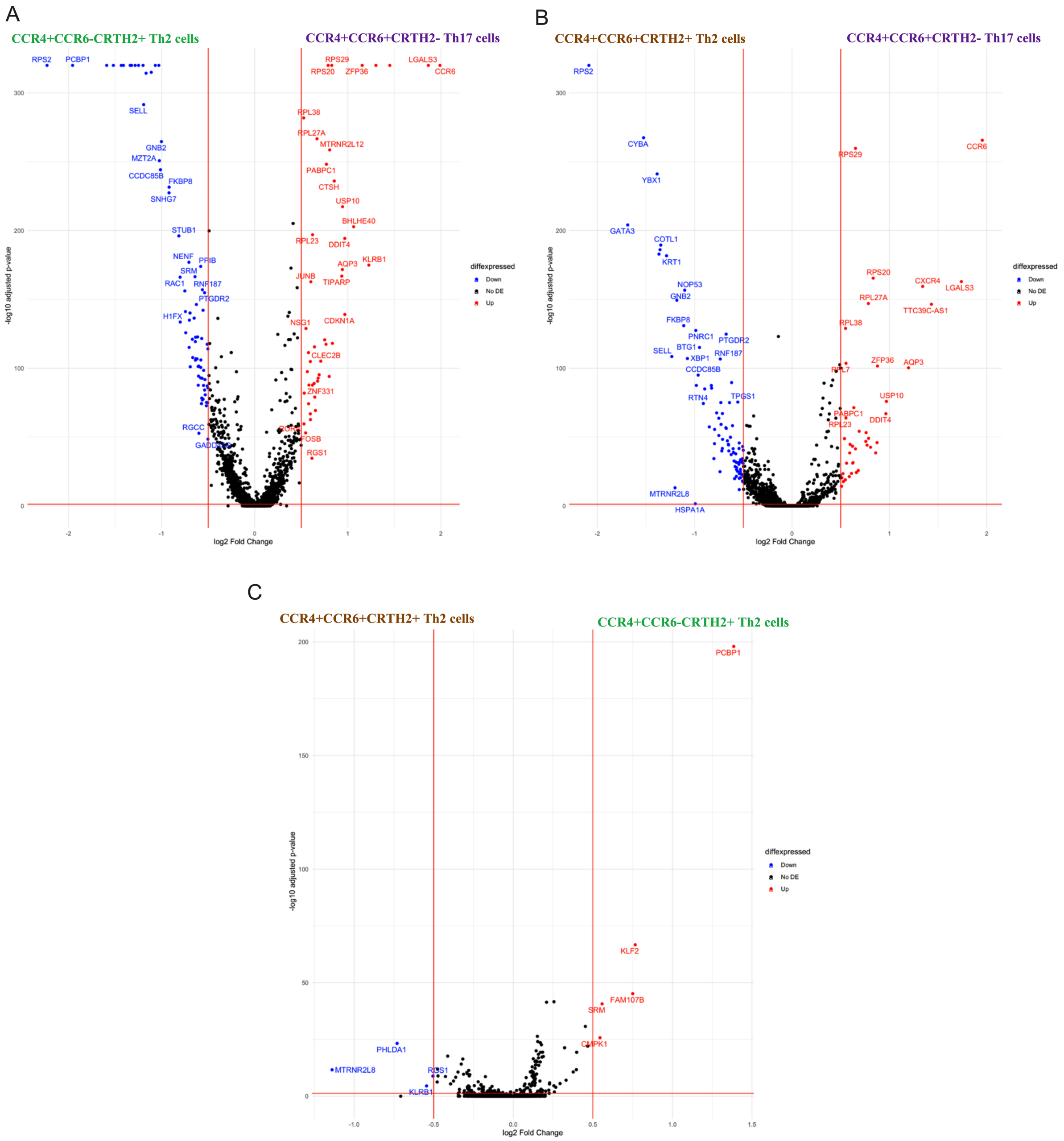

Supplemental figure 5: Molecular signature of the three filarial-enriched T_{EM} $CD4^+$ T cells subsets. Volcanos plots highlight the differentially expressed genes for each DICE-annotated T_{EM} $CD4^+$ T cells subsets, including $CCR4^+CCR6^+CRTH2^-$ Th17 cells *vs.* $CCR4^+CCR6^-CRTH2^+$ Th2 cells (A), $CCR4^+CCR6^+CRTH2^-$ Th17 cells *vs.* $CCR4^+CCR6^+CRTH2^+$ Th2 cells (B), and $CCR4^+CCR6^-CRTH2^-$ Th2 cells *vs.* $CCR4^+CCR6^+CRTH2^+$ Th2 cells (C).

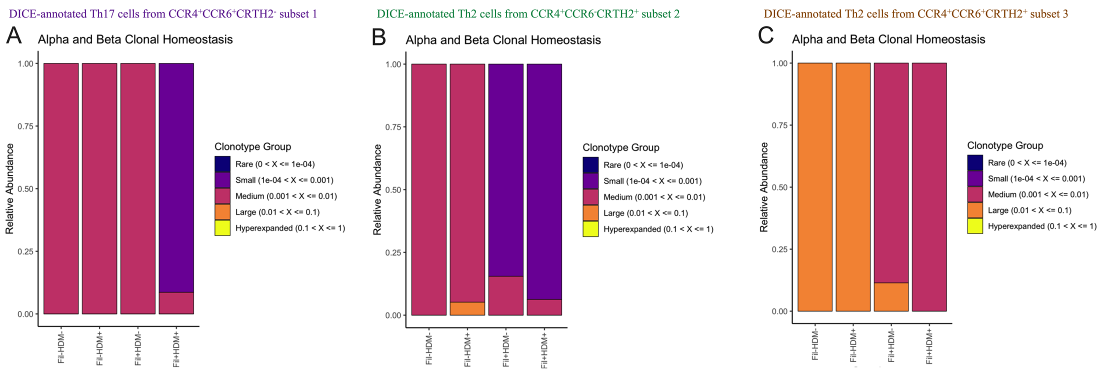

Supplemental figure 6. Alpha and Beta clonal homeostasis. TCR analysis using scRepertoire package highlighting the clonal homeostasis of the clonotypes within each DICE-annotated effector memory CD4+ T cell subset. Different colors in the graphs indicates the relative abundance ordered by the clonotype proportions.

Supplemental table 1: Flow cytometry antibodies for immunophenotypic analysis

Detector	Fluorochrome	Ab specificity	Clone	Catalog #	Source
B515	FITC	CRTH2	BM16	561659	BD Pharmigen
B710	BB700	OX40	ACT35	566560	BD Horizon
V450	BV421	IL-5	TRFK5	504311	BioLegend
V510	BV510	CCR4	L291H4	359416	BioLegend
V570	BV570	CD8	RPA-T8	301038	BioLegend
V605	BV605	IL-4	MP4-25D2	500828	BioLegend
V655	BV650	CXCR3	1C6	740603	BD OptiBuild
V710	BV711	CD137	4B4-1	569689	BD Horizon
V750	BV750	TNF	MAb11	566359	BD Horizon
V785	BV785	CD127	A019D5	351330	BioLegend
R670	APC	CD154	24-31	17-1548-42	eBioscience
R730	AF700	CCR7	G043H7	353244	BioLegend
R780	APC/Cy7	CXCR5	J252D4	356926	BioLegend
U390	BUV395	IFNg	B27	563563	BD Horizon
U450	Live/Dead-UV	Viability	--	L34961	Invitrogen
U500	BUV496	CCR6	11A9	612948	BD Horizon
U570	BUV563	CD45RA	H100	612927	BD Horizon
U660	BUV661	CD3	UCHT1	612965	BD Horizon
U740	BUV737	PD-1	EH12.1	612791	BD Horizon
U785	BUV805	CD4	SK3	612888	BD Horizon
G575	PE	IL-10	JES3-9D7	12-7108-82	eBioscience
G610	PE/Dazzle 594	TCRgd	B1	331226	BioLegend
G660	PE/Cy5	CD25	BC96	302608	BioLegend
G710	PE/Cy5.5	CD69	CH/4	MHCD6918	Invitrogen
G780	PE/Cy7	IL-13	JES10-5A2	501914	BioLegend

Supplemental table 2: Study population

Group / Patient ID	Diagnosis	Phadiatop (kUA/L)	IgE-specific to... (kUA/L)					Total IgE
Atopic+Filaria+			*Dermatophagoides pteronyssinus*	Timothy Grass Pollens	German cockroach	cat dander	*Alternaria alternata*	
1	*Loa loa*	22.5	11.8	10.8	27.5	0.4	0.45	497.0
2	*Wuchereria bancrofti*	5.41	13.30	0	0	0	0	120.0
3	*Onchocerva volvulus*	48	16.1	28.7	1.64	0	0	755.0
4	*Loa loa*	43.4	38.50	14.80	7.18	0.56	0	1244.0
5	*Loa loa*	5.34	4.82	2.27	5.79	1.33	0	9296.0
6	*Loa loa*	26.4	2.3	4.42	11.8	0	0.7	11794.0
7	*Loa loa/Onchocerca volvulus*	30.8	45.7	3.02	1.59	1.62	0	1269.0
8	*Loa loa*	4.12	3.22	0.47	2.49	0.40	0.36	6206.0
9	*Loa loa*	1.39	0.58	0	1.15	0	0	606.0
10	*Loa loa*	35.7	2.05	10.6	3.65	0	1.8	1774.0
11	*Loa loa*	1.35	0.65	0	0.43	0	0.43	1506.0
12	*Loa loa*	4.38	1.01	3.65	4.31	0	0	
**Atopic-Filaria+**								
1	*Loa loa*	0						43.3
2	*Loa loa*	0						115.0
3	*Loa loa*	0						187.0
4	*Loa loa*	0						33.9
5	*Loa loa*	0						2794.0
6	*Loa loa*	0						106.0
7	*Onchocerca volvulus*	0						172.0
8	*Wuchereria bancrofti*	0						99.2
9	*Loa loa*	0						787.0
10	*Wuchereria bancrofti*	0						26.0
11	*Loa loa*	0						28.0
**Atopic+Filaria-**								
1	Negative	21.9	3.47	1.79	4.58	1.78	9.23	457
2	Negative	5.58	2.64	0	3.74	0	0	107.00
3	Negative	11.9	8.73	1.45	0	0	0	44
4	Negative	11.1	0.8	6.5	0.4	0.0	0.0	35.20
5	Negative	24.1	5.65	12.2	0	0.61	0	74.30
6	Negative	13.7	1.24	6.92	0	1.16	1.44	283.00
7	Negative	7.68	6.78	6.92	0	0	0	10.73
8	Negative	26.6	8.13	3.12	0	0	0	60.09
9	Negative	5.56	2.59	0	2.1	0	0	18.40
10	Negative	16.2	4.26	3.04	4.48	0	0	57.40
11	Negative	14.5	2.1	0	11.3	0	2.16	121.82
12	Negative	39.8	9.44	4.85	15.6	2.6	16.4	191.1
**Atopic-Filaria-**								
1	Negative	0						5.62
2	Negative	0						8.35
3	Negative	0						2.37
4	Negative	0						9.70
5	Negative	0						8.94
6	Negative	0						24.3
7	Negative	0						2
8	Negative	0						143.00
9	Negative	0						7.1
10	Negative	0						81.10
11	Negative	0						2.59
12	Negative	0						3.08

Supplemental table 3: Flow cytometry antibodies for sorting

Detector	Fluorochrome	Ab specificity	Clone	Catalog #	SOURCE
B515	FITC	CRTH2	BM16	561659	BD Pharmigen
V450	BV421	CCR4	L291H4	359414	BioLegend
V510	BV510	Aqua Live/Dead	--	L34957	Invitrogen
R730	AF700	CCR7	G043H7	353244	BioLegend
U500	BUV496	CCR6	11A9	612948	BD Horizon
U660	BUV661	CD3	UCHT1	612965	BD Horizon
G610	ECD	CD45RO	UCHL1	IM2712U	Beckman Coulter
G780	PE/Cy7	CD4	OKT4	317414	BioLegend
